# Stacking Pressure‐Driven Interfacial Dynamics in Anode‐Free Solid‐State Lithium Batteries

**DOI:** 10.1002/advs.76888

**Published:** 2026-08-05

**Authors:** Jianneng Liang, Matthias Bohnen, Ralf Müller, Robert Leiter, Simon Fleischmann, Shuya Gong, Mervyn Rohit Soans, Stefano Passerini, Alberto Varzi

**Affiliations:** ^1^ School of Resources Environment and Materials Guangxi University Nanning China; ^2^ Helmholtz Institute Ulm (HIU) Electrochemical Energy Storage Ulm Germany; ^3^ Karlsruhe Institute of Technology (KIT) Karlsruhe Germany; ^4^ Institute for Mechanics Department of Civil and Environmental Engineering Technical University of Darmstadt Darmstadt Germany; ^5^ International Electrochemical Energy Storage Research Institute (IEES) School of Energy Science and Engineering Nanjing Normal University Nanjing China

**Keywords:** anode‐free solid‐state batteries, interface, lithium dendrite, reference electrode

## Abstract

Stacking pressure plays a critical role in maintaining the electrochemical performance of solid‐state batteries (SSBs), including anode‐free solid‐state batteries (AFSSBs). Nevertheless, the influence of stacking pressure on interface properties remains insufficiently understood. In this work, we found that stacking pressure could improve both anode and cathode interface electrochemical properties, but the enhancement of the cathode side is considerably smaller than that observed at the anode interface, indicating that pressure primarily benefits the anode side. We also establish a correlation among stacking pressure, anode and cathode potentials, interface resistances, Li deposition morphology, and stress distribution in AFSSBs. Our results show that increasing the stacking pressure leads to higher reversible capacity, lower Li plating/stripping overpotentials, more uniform Li deposition, and a more homogeneous stress distribution. Achieving uniform Li deposition is key to reducing the required magnitude of stacking pressure. This study deepens the understanding of interfacial dynamics in AFSSBs and paves the way toward developing high‐performance SSBs operable under low stacking pressure.

## Graphical abstract

Using a three‐electrode setup, it is found that stacking pressure improves both anode and cathode interfaces but more effectively stabilizes the anode. Under low pressure, a sudden anode resistance rise indicates contact loss. Higher pressure delays this via Li creep and plastic deformation, maintaining interfacial contact. The cathode shows minor changes, confirming that pressure primarily benefits the anode interface.

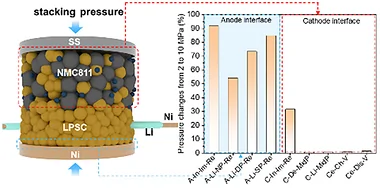

## 1 Introduction

The widespread adoption of portable electronics, electric vehicles, and electric aircraft, among others, has recently increased the demand for safe and high‐energy‐density storage devices. State‐of‐the‐art lithium‐ion batteries (LIBs) struggle to keep up with such increasing standards because of their limited energy density and inherent safety issues. Many advanced battery configurations were therefore developed and investigated in recent years. Among them, lithium metal batteries with anode‐free (or anode‐less) configuration and solid‐state electrolytes (SSEs) are among the most promising candidates for next‐generation energy storage devices [1, 2, 3, 4, 5, 6]. As the name suggests, anode‐free solid‐state batteries (AFSSBs) lack excess anode active material and flammable liquid organic electrolyte, which could effectively improve both the energy density and the safety [7, 8, 9, 10]. Besides, unlike conventional Li metal batteries (LMBs) where metallic Li is handled during the battery assembling process, the production of AFSSB is much simpler. This further improves safety and reduces manufacturing costs [7, 9].

Despite the aforementioned benefits, there are still several critical challenges that hinder the development of AFSSBs, as well as Li metal‐based solid‐state batteries (SSBs). These challenges are correlated to the Li plating/stripping behavior at the negative electrode. First, the uneven Li plating and stripping behavior will damage the integrity of the current collector (CC) and SSE interface, forming uncontacted areas and leading to the formation of dead Li, lowering the Coulombic efficiency [7, 11]. Furthermore, the formation of highly reactive metallic Li during charge leads to side reactions with both SSE and CC, also resulting in the loss of active Li and low Coulombic efficiency. The Li plating and stripping process also causes a volume change of the solid‐state cell. For example, in an AFSSB with 3 mAh cm^−2^ cathode active material (CAM) loading, theoretically, a 14.6 µm thick Li metal layer is deposited between CC and SSE, considering the Li deposit to be 100% dense [12]. The resulting volume expansion will cause pressure build‐up inside the cell, while the following Li stripping may induce the formation of voids and gaps at the interface, as the pressure is released. Altogether, these issues will lead to the degradation of interface contact, resulting in low Coulombic efficiency and short cycling life of the AFSSB. These processes are referred to as interfacial electro‐chemo‐mechanical phenomena in SSBs.

The electro‐chemo‐mechanical properties of SSBs require stacking pressures to maintain good interface contact and to ensure good electrochemical performance. Kasemchainan et al. [13] investigated the plating/stripping behavior of Li|Li_6_PS_5_Cl|Li symmetric cells under different stacking pressures, disclosing that the critical stripping current is a major factor limiting the electrochemical performance of SSB. Doux et al. [14] optimized the stacking pressure for Li|Li_6_PS_5_Cl|Li symmetric cells as well. They demonstrated that very high stacking pressures (>75 MPa) will mechanically short the cell due to Li creep, causing severe cracking of SSE. Chang et al. also found a creep‐dominated Li deposition behavior of the Li/Li_7_La_3_Zr_2_O_12_ interface above 7.4 MPa stacking pressure [15]. Nonetheless, plenty of studies about stacking pressure effects in SSBs with Li metal anode have reached the conclusion that an optimized stacking pressure plays a very critical role in improving the electrochemical performance of SSBs [16, 17, 18, 19, 20].

The effect of stacking pressure has been investigated in anode‐free batteries as well, but to a much lesser extent. Lin et al. [21] used in situ solid‐state nuclear magnetic resonance (ss‐NMR) to study the influence of stacking pressure on dendritic behavior and dead Li formation in liquid electrolyte‐based anode‐free cells. At 0.1 MPa, Li dendrites rapidly formed, followed by a linear increase in dead Li. Higher stacking pressure not only causes Li metal to fracture, but also leads to the formation of dendrites and dead Li at the fracture site. Kazyak et al. [22] used operando three‐dimensional (3D) video microscopy to demonstrate the interplay between electrochemical behavior and mechanics at the interface between a garnet SSE (Li_6.5_La_3_Zr_1.5_Ta_0.5_O_12_) and Cu‐CC during Li deposition. They developed a model to describe the progression of Li plating from nucleation to growth of Li blisters at the interface. They found that the stacking pressure, the adhesive forces between CC and SSE, and the chemical and mechanical properties of CC, such as thickness, elastic and plastic properties, all play roles in determining the Li deposition morphology.

Critically, the influence of stacking pressure differs markedly between solid‐state Li metal battery (SSLB) and AFSSB systems. In SSLMBs, Li directly deposits at the interface between Li and SSE, failure arises from interfacial voids and Li dendrite penetration; stacking pressure aids Li deformation and contact maintenance. In AFSSBs, the primary issues are poor CC/SSE contact uniformity, current crowding, and lithiophobic CC surfaces, all of which cause uneven Li deposition/stripping and interfacial disconnection. Here, the stacking pressure ensures intimate CC/SSE contact, lowers the Li nucleation barrier, and deforms deposited Li to enable uniform layered growth. Findings and theories established in SSLBs cannot be directly applied to anode‐free lithium batteries. In AFSSB, there are still critical questions remaining unanswered. For example, whether stacking pressure exerts a positive effect on both the anode and the cathode interfaces and, if so, which interface is more affected. This issue remains unresolved primarily due to the lack of suitable characterization techniques capable of independently investigating anode and cathode interfaces in SSBs.

To address this problem, herein, we developed a 3‐electrode (3‐e) AFSSB cell setup, allowing to separately monitor the anode and cathode potentials versus a reference electrode (RE), as well as to record single interface impedance. Different stacking pressures were applied to the AFSSBs, and their effects on the electrochemical performance and Li deposition morphology were investigated systematically. By combining electrochemical testing, mechanical simulation, and advanced materials characterization techniques, we quantified the stacking pressure asymmetric effect at the anode and cathode interfaces. We found that stacking pressure exerts positive effects on both the anode and cathode interfaces, as well as on the associated charge transfer processes. Nevertheless, stacking pressure plays a more critical role at the anode interface, owing to the lower yield modulus of Li metal and the larger volume change of the Li anode. Unfortunately, stacking pressure cannot be increased indefinitely to enhance electrochemical performance; a high stacking pressure of up to 20 MPa leads to rapid short‐circuiting, caused by the creep of Li metal and its subsequent penetration into SSE. In this study, we established correlations between stacking pressure and multiple performance metrics—including overpotential, impedance, stress distribution, and Li deposition uniformity—to elucidate the mechanistic role of stacking pressure in AFSSB performance and failure modes.

## 2 Results and Discussion

To study the effect of stacking pressure on the electrochemical performance of AFSSBs, a 2‐electrode (2‐e) configuration AFSSB was first used to evaluate the overall electrochemical response under different conditions. Most of the previous works focused on the negative electrode and used either symmetric Li|SSE|Li or asymmetric Li|SSE|current collector (CC) cells for this purpose [13, 14, 22]. Herein, an anode‐free CC|SSE|LiNi_0.8_Mn_0.1_Co_0.1_O_2_ (NMC811) full cell was employed instead, in order to replicate conditions closer to the practical application. As described in the Methods section, AFSSBs were first densified under 370 MPa pressure. Afterwards, the stacking pressure was reduced to the targeted values (0, 2, 5, 10, and 20 MPa) before undergoing electrochemical testing.

The impedance of fresh 2‐e AFSSBs decreased from ∼2000 to 50 Ω (high‐frequency, end of the first semicircle) with stacking pressures increasing from 0 to 2, 5, 10, and 20 MPa (Figure S1). A similar trend could be observed from the voltage‐capacity profiles (Figure S2). With no stacking pressure applied (0 MPa), AFSSB showed a high charging voltage followed by a sudden voltage drop when the capacity exceeded 30 mAh g^−1^, indicating short circuiting of the cell [23]. This confirms how challenging it is to achieve good electrochemical performance with a stacking pressure‐free AFSSB. At 2 MPa, the cell could be successfully charged to the target cut‐off voltage of 4.25 V, but the large overpotential led to a relatively low capacity (102 mAh g^−1^ in the following first discharge cycle). When the stacking pressure was increased to 5 MPa, the discharge capacity increased to 131 mAh g^−1^ and the overpotential decreased significantly. At 10 MPa, an even higher discharge capacity (139 mAh g^−1^) could be obtained; however, with only a minor decrease in the overpotential compared to 5 MPa. Further increasing the stacking pressure to 20 MPa led to neither higher capacity nor lower polarization. Moreover, in the following cycles, overcharging occurred, suggesting the occurrence of soft short‐circuits of the cell (Figure S3) [23, 24].

Afterwards, a 3‐e solid‐state cell was employed to deconvolute the response from anode and cathode. The cell includes a Li‐coated Ni‐wire RE, a Ni CC, and a NMC811+Li_6_PS_5_Cl (LPSC)+carbon composite cathode (schematically illustrated in Figure 1a). The RE allows recording both the potential and impedance response of the individual electrodes, thereby enabling a more accurate study of the anode and cathode interfaces (Figure S4) [13, 25, 26, 27, 28, 29]. Figure 1b displays the cell voltage and cathode potential vs. capacity profiles of 3‐e cells under 2 and 10 MPa stacking pressure. Here, we observed that increasing stacking pressure from 2 to 10 MPa lowers the charging profile for both the cell voltage and cathode potential during cycling, indicating reduced polarization and improved cathode–electrolyte interfacial properties. The 10 MPa stacking pressure also improved the discharge capacity of AFSSBs from ∼102 to 139 mAh g^−1^. For the anode side (Figure 1c), the Li nucleation potential decreased from 48 mV vs. Li/Li^+^ to 22 mV vs. Li/Li^+^ going from 2 to 10 MPa. The Li plating (deposition) and stripping potentials at 10 MPa stacking pressure were rather stable at ‐8 and 4 mV vs. Li/Li^+^, respectively, compared to ‐30 and 26 mV vs. Li/Li^+^ for the cell subjected to 2 MPa.

**FIGURE 1:**
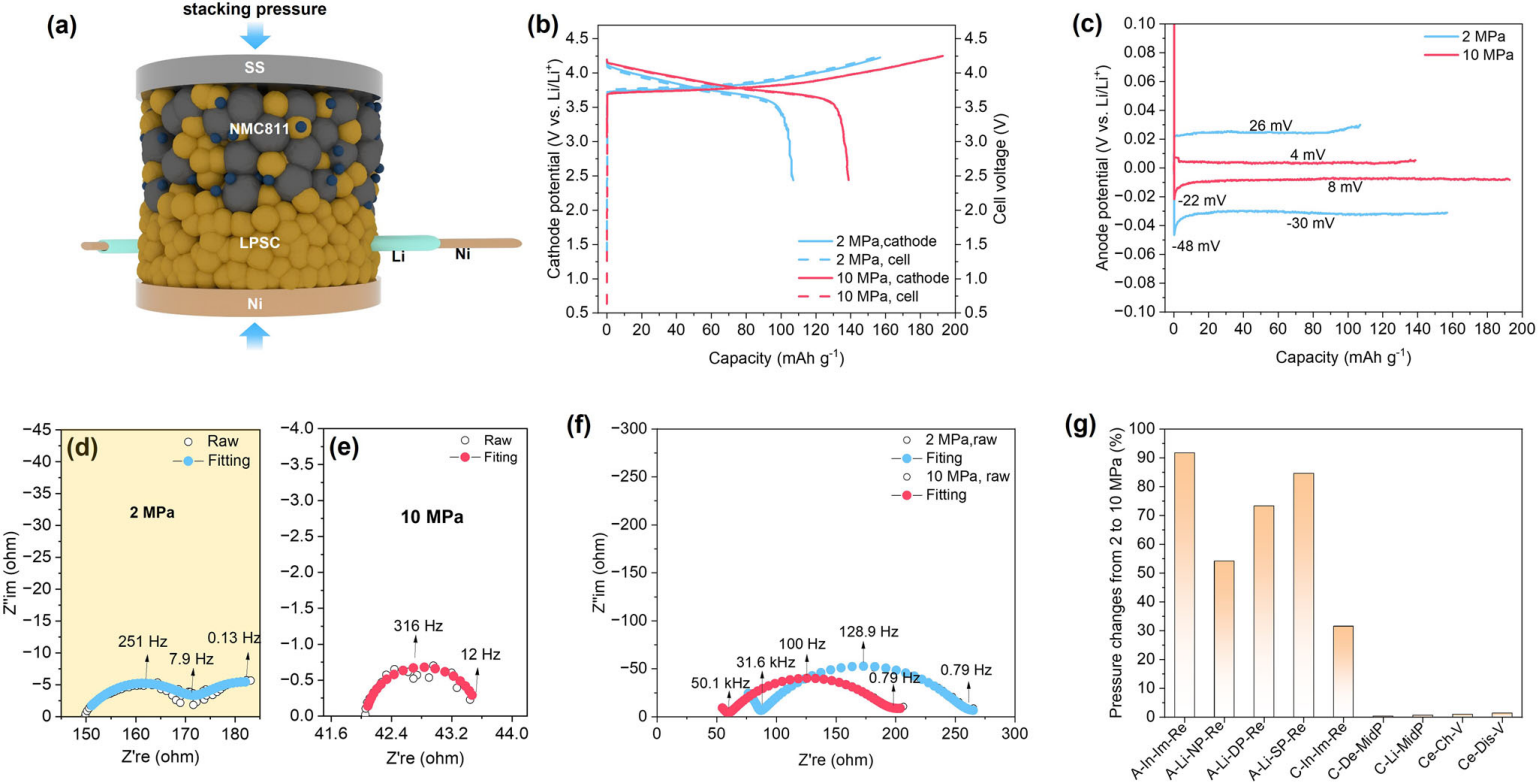
(a) Schematic diagram showing the 3‐e AFSSB configuration and the applied stacking pressure. (b) Cathode potential and cell voltage vs. capacity profiles. (c) Anode potential vs. capacity profiles. Nyquist plots of (d, e) anode and (f) cathode impedance spectra at the end of charge state at 2 and 10 MPa stacking pressure. (g) Comparisons of the reduction ratios of interface impedances, potential, and cell mid‐voltages upon increasing the stacking pressure from 2 to 10 MPa.

Using the 3‐e cell setup, impedance spectra of anode, cathode, and full cell could also be collected simultaneously. Figure 1d,e illustrates the Nyquist plots of the anode at the end of the first charge (i.e., after Li plating on the current collector). Spectra were fitted with an equivalent circuit (Figure S5) including the SSE resistance (R*_SSE_*) in series with two (R | C) nets, i.e. a (R*_SEI/CEI_* | CPE*_SEI/CEI_*) net and a (R*_ct_* | CPE*_ct_*) net. R*_SEI/CEI_* and CPE*_SEI/CEI_* represent the resistance and capacitance associated with the solid electrolyte interphase (SEI) or cathodic electrolyte interphase (CEI), while R*_ct_* and CPE*_ct_* represent the resistance and capacitance associated with the charge transfer. The 2 MPa impedance spectrum presents two distinguished semi‐circles corresponding to (R*_SEI/CEI_* | CPE*_SEI/CEI_*) net and a (R*_ct_* | CPE*_ct_*) net, while these two semi‐circles merge into one at 10 MPa, possibly due to the smaller impedance associated with charge transfer. At 10 MPa stacking pressure, both anode and cathode interface resistances (R*_SEI/CEI_* + R*_ct_*) were smaller than those at 2 MPa, demonstrating improved interfacial properties. The interface resistance at the cathode side decreases from 177.7 to 121.5 Ω with the stacking pressure increasing from 2 to 10 MPa, corresponding to a 31% decrease (Figure 1f). However, the resistance at the anode decreases from 18.1 to 1.5 Ω with stacking pressure increasing from 2 to 10 MPa, i.e., a 92% decrease (Figure 1f). We further compared the Li nucleation potentials, Li deposition and stripping potentials, cathode mid‐potentials, and cell mid‐voltages under 2 and 10 MPa. The results are presented in Figure 1g. It shows that 10 MPa stacking pressure improves all the above electrochemical parameters. The enhancement is quantified using an improvement ratio, defined as the percentage increase in a parameter relative to the corresponding value at 2 MPa. It is found that the anode interface impedance reduction ratio (A‐In‐Im‐Re) is more than 90%, the anode Li nucleation potential reduction ratio (A‐Li‐NP‐Re) is approximately 52%, the anode Li deposition potential reduction ratio (A‐Li‐DP‐Re) is around 75%, and the anode Li stripping potential reduction ratio (A‐Li‐SP‐Re) is over 85%. However, on the cathode side, except for the cathode interface impedance reduction ratio (C‐In‐Im‐Re) being around 30%, both the cathode delithiation mid‐potential reduction ratio (C‐De‐MidP) and cathode lithiation mid‐potential increase ratio (C‐Li‐MidP) improvements are smaller than 2%. Besides, cell charging mid‐voltage reduction ratio (Ce‐Ch‐V) and cell discharging mid‐voltage increase ratio (Ce‐Dis‐V) are also smaller than 2%. The largest improvements were only related to the anode interface/Li deposition and stripping processes. These results suggest that stacking pressure has a significantly greater impact on the anode CC/SSE interface rather than on the cathode/SSE interface.

Figure 2a,d shows the evolution of electrode potentials and cell voltage of 3‐e AFSSBs at 2 and 10 MPa. The cycling life of AFSSBs with different stacking pressures is presented in Figure S6. The initial anode potential for the freshly assembled AFSSB was around 2 V (Figures S7 and S8), which is expected for Ni CC vs. Li/Li^+^. Once charging starts, the anode potential drops to ∼0 V quickly because of Li metal deposition on the Ni CC. If all the deposited Li could be entirely stripped during discharge, the anode potential would return to around 2 V vs. Li/Li^+^. However, during initial cycles, at the end of each discharge step (Li stripping from the anode), the cell voltage had already dropped to 2.5 V (discharging cut‐off voltage), while the anode potential remained close to 0 V. This suggests that the Li stripping step was terminated before complete removal of Li from the Ni CC. Therefore, the residual Li at the CC/SSE interface results in an anode potential of ∼0 V vs. Li/Li^+^ at the resting state. However, after a certain number of cycles, a sudden increase in anode potential (Figure 2a,b and Figures S7 and S8 insert) is observed before the cathode potential reaches its target value, leading to a quick drop in the cell voltage to the discharging cut‐off voltage. This results from insufficient active Li at the anode side, possibly due to the formation of dead Li and side reaction products, among others [30, 31, 32].

**FIGURE 2:**
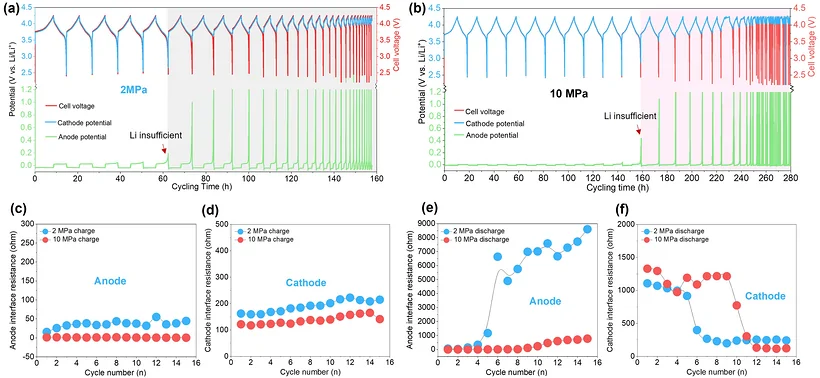
Electrode potentials and cell voltage vs. time profiles of 3‐e AFSSBs at (a) 2 MPa, and (b) 10 MPa stacking pressure; (c) and (d) show the evolution of anode and cathode interface resistance evolutions at the end of charge; and (e) and (f) are the anode and cathode interface resistance evolutions at the end of discharge.

The evolution of impedance data collected in 3‐e mode also provides insights into the origin of the anode potential increase. As shown in Figure 2c,d, at the end of charge (Li plating), both anode and cathode interface resistances are relatively stable upon cycling, with small values (tens to 200 Ω). However, at the end of discharge, the anode's interface resistance is stable only until a sudden increase in the anode potential is observed during cycling (fifth cycle). In fact, the cell subjected to 2 MPa (Figure 2e), shows a sudden increase in the anode interface resistance to several kΩ from the fifth cycle, corresponding to the increase of the anode potential vs. time profile (Figure 2a). These abrupt and large changes in anode interface resistance strongly point out the loss of interfacial contact. Similarly, for the cell subjected to 10 MPa, the anode interface resistance remains stable within the first 10 cycles, consistent with the cycle at which the anode potential starts increasing (Figure 2b). In this case, the anode interface resistance increases to “only” several hundred Ω, approximately one order of magnitude lower than that observed at 2 MPa. Interestingly, the cathode interface resistance evolves in the opposite manner. At the end of discharge, the cathode interface resistance decreases from ∼1100 to ∼200 Ω for 2 MPa, and from ∼1200 to ∼100 Ω for 10 MPa. These results suggest that, again, the stacking pressure has a much stronger influence on the anode interface and only a minor effect on the cathode interface.

For completeness, the full cell impedance spectra (2‐e cell) of the AFSSBs are reported in Figures S9 and S10. These spectra were also processed to obtain the distribution of relaxation times (DRT), which allows us to distinguish the response of the different system components from the response of the system as a whole [33, 34, 35]. Six distinct peaks (P1‐P6) were obtained after DRT simulation (Figure 3), each corresponding to key impedance components, i.e., grain boundary resistance of SSE (P1) [33, 36], solid‐solid contact among particle (P2) [36], Li‐ion diffusion across SEI (P3) [37], Li‐ion transportation across CEI (P4) [37], and charge transfer (P5, P6) [37], respectively. An obvious increase in P3 intensity could be observed for AFSSB cycled at 2 MPa at the end of charge state (Figure 3a), suggesting that the interphase between SSE and anode (SEI) worsens upon cycling. At the end of discharge, all peaks’ intensities increase significantly after 5 cycles (Figure 3b), suggesting the failure of the AFSSB arises from multiple aspects, including SSE grain boundary, powder contact, Li‐ion diffusion across SEI/CEI, and charge transfer processes.

**FIGURE 3:**
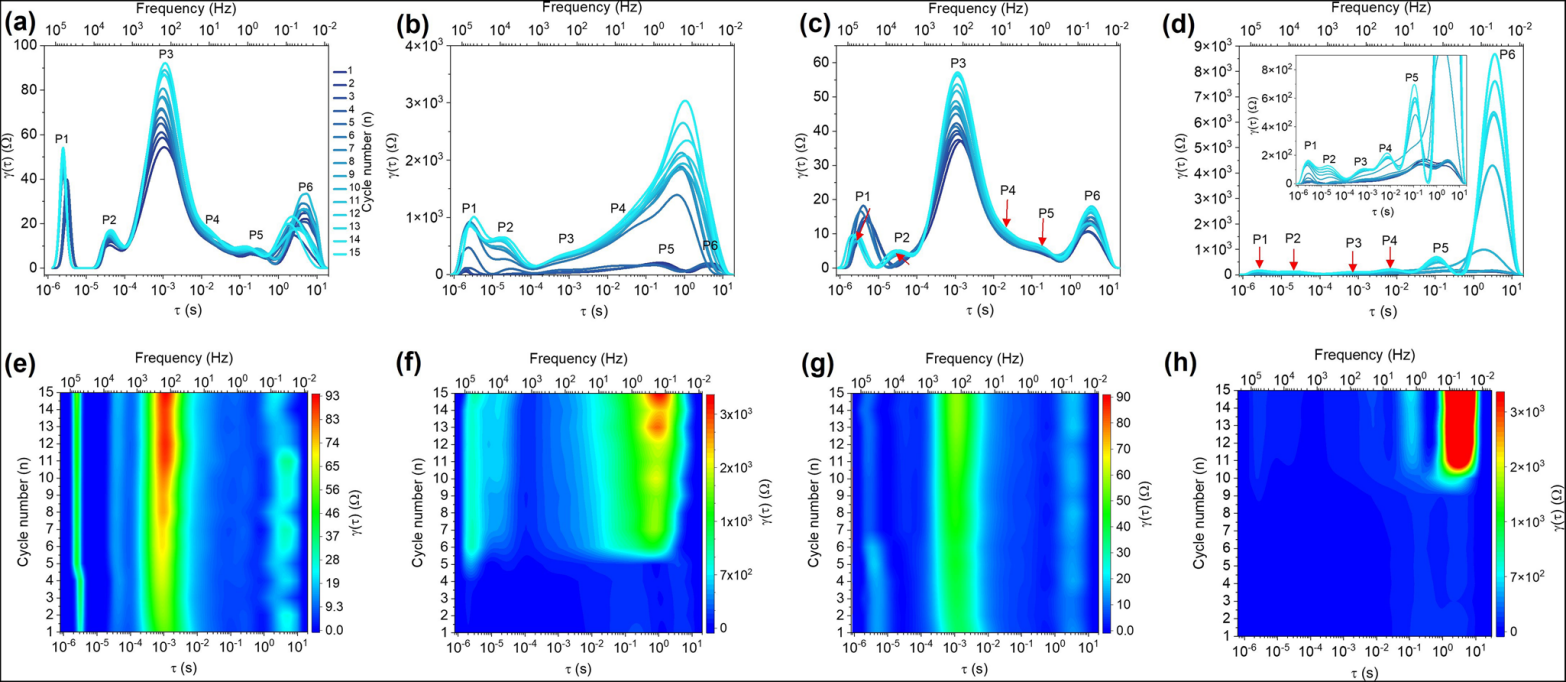
Evaluation of DRT responses for AFSSBs at the end of charge state at (a) 2 MPa, and (c) 10 MPa, and at the end of discharging state at (b) 2 MPa and (d) 10 MPa. (e) to (h) are the corresponding 2D intensity maps of (a) to (d).

Similarly, for the AFSSB cycled under 10 MPa, the intensity of P3 increased noticeably (Figure 3c), indicating instability of the SEI layer. In contrast, the intensity of P1 decreased, which may be attributed to the formation of an additional Li layer at the anode interface. This layer could increase the internal pressure of the SSB, thereby reducing the grain boundary resistance of the SSE. At the end of discharge, all peak intensities showed a clear increase after 10 cycles (Figure 3d), consistent with the trend observed under 2 MPa. Notably, the intensity increments were more pronounced for P5 and P6, both associated with charge transfer processes. The corresponding 2D intensity color maps presented in Figure 3e–h further illustrate the evolution of these peak intensities with cycling for AFSSBs under both 2 and 10 MPa stacking pressures.

To further explore how stacking pressure influences the anode interface, Li deposition behavior and mechanical properties of the interfaces were investigated by Cryo‐TEM (Figure S11). Figure S11a,b illustrates the cryo‐TEM images of Li deposited at the Ni/SSE interface (for this specific case, a Ni TEM grid was used as a current collector), where an island‐like Li metal growth can be clearly seen. Bright‐field and dark‐field TEM images of the Li island were then compared. Under dark‐field‐TEM, the presence of bright nanoparticles on the Li island's surface could be detected. Using electron diffraction (Figure S11c), we could identify these nano‐particles being Li_2_O. Although the whole workflow was done under an inert environment to avoid contamination of Li metal, we cannot exclude this being caused by reaction with O_2_ traces in the glovebox during TEM sample preparation. EELS was performed on the top surface of this Li island, as indicated in the circular area in Figure S11d. The corresponding Li EELS spectrum (Figure S11e) presents the characteristic response of metallic Li at 55 eV, suggesting that this rod is constituted from Li metal [38]. The additional peak at 63 eV may originate from Li_2_O [39], since we also observed it from the electron diffraction pattern (Figure S11c). In addition, no S EELS peak was found in this area (Figure S11f). This might be due to the fact that, in SSBs, Li deposits under the SEI, so no new interphase is generated at the newly formed Li surface. The SEI in contact with the SSE may include SSE‐derived species (Li_2_S, Li_3_P, LiCl), but the SEI on bulk Li surface (not touching SSE) should lack these. To prove this, EELS and EDX mapping were performed on a Li rod in contact with the SSE. From the EELS mapping (Figure S12a–c), the distribution of Li metal could be observed. From the EDX mapping (Figure S12d,e), it is found that S is concentrated on the SSE particle, whereas O is concentrated on Li metal. No S could be detected on the Li metal rod. This confirms that the Li_2_O is not part of the SEI between Li and SSE, but rather a side‐product resulting from Li reactivity with O_2_/H_2_O traces.

The SEM images in Figure 4a,c and Figure S13 show the CC surface at the end of charge upon different stacking pressures. At 0 MPa, the size of Li islands on the CC surface ranges between 5 and 50 µm (Figure S13a). The deposition area and the size of Li islands on the CC surface are rather non‐uniform. With 2 MPa stacking pressure, the size of Li islands visibly increases between 100 and 500 µm (Figure 4a and Figure S13b). The distribution of Li islands on the CC surface also becomes relatively more uniform. With 5 MPa stacking pressure, the CC surface is almost covered by Li islands, with only a small area without Li deposition (Figure S13c). Moreover, the growing Li islands begin merging together. With 10 MPa, the coverage of the CC surface is almost complete (Figure 4c and Figure S13d). The deposition of Li islands looks more like a dense layer on the CC surface. With 20 MPa, the coverage of the CC surface is a totally complete (Figure S13e), indicating a more even Li deposition behavior with higher stacking pressure. The schematic diagrams under the SEM images (Figure 4e,g and Figure S13e–i) show the growth mechanism of Li islands at different pressures. The formation of uniform Li deposition layers under high stacking pressure is the result of many Li islands growing up and merging together. This can be clearly appreciated by looking at the temporal evolution of the CC surface during Li plating (Figure S14).

**FIGURE 4:**
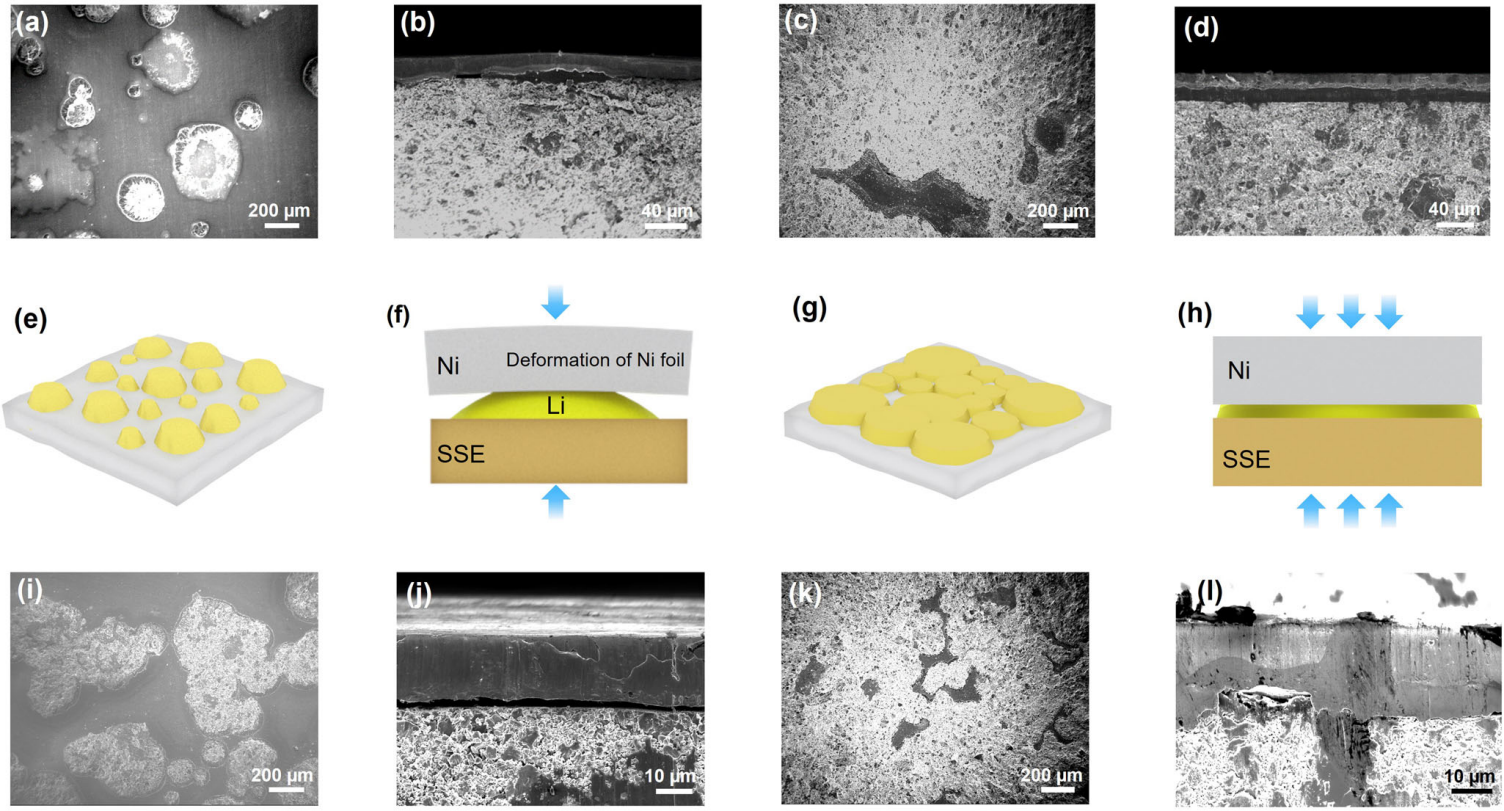
(a) surface and (b) cross‐section SEM images of Ni CC after first charging the AFSSBs at 2 MPa; and (c), (d) at 10 MPa. (e–h) are their corresponding schematic diagrams. (i) surface and (j) cross‐section SEM images of Ni CC after first discharging the AFSSBs at 2 MPa; and (k), (l) at 10 MPa.

These results indicate that deposited Li experiences creep behavior, forming a flat deposit. Higher stacking pressure results in more significant creep and flatter Li deposition. This is confirmed by the cross‐section SEM image of Li. With 2 MPa stacking pressure, the growth of Li island at the interface clearly deforms the Ni CC, forming a bump (Figure 4b). This process, schematically illustrated in Figure 4f, is consistent with the work published by Motoyama et al. [40] and Krauskopf et al. [41], reporting that when the CC is not sufficiently strong to withstand the stress induced by the growth of a Li island, it will swell and break. Under a high stacking pressure (10 MPa), due to the soft characteristic of Li metal, the island is deformed rather than CC, resulting in an even Li deposition morphology at the CC/SSE interface (Figure 4d). This process is schematically illustrated in Figure 4h.

Besides the above‐discussed Li deposition behavior, we found another issue at the CC/SSE interface. A broad view of the CC/Li/SSE interface was investigated by FIB‐SEM. Figure S15 displays the cross‐sectional view of the CC/Li/SSE interface from an AFSSB at 10 MPa at the end of charge. The results suggest that, due to the particulate nature of the SSE, Li deposits at the CC/SSE interface tend to encapsulate adjacent SSE particles within the growing Li layer. This encapsulation can compromise interfacial integrity. Specifically, it may degrade Li/CC contact and induce non‐uniform current distribution, as SSE particles inherently exhibit poor adhesion to the CC. Application of high stacking pressure, however, can help preserve interfacial contact and prevent such detachment.

At the end of the discharging state (Li stripping from Ni CC), we observe Li metal adherent to the CC surface (Figure 4i). This means that residues of Li are left on the CC/SSE interface after stripping. This is where Li losses originate. In addition, the CC loses contact with the SSE (Figure 4j), meaning the stacking pressure is insufficient to achieve intimate contact at the interface. In contrast, at 10 MPa, the Ni CC surface was fully covered by LPSC SSE (Figure 4k), and CC appeared to be in intimate contact with SSE (Figure 4l). We further employed focused ion beam (FIB) to explore the morphology of the buried CC/SSE interface at the end of discharge, as presented in Figure S16. It is evident that Li nucleation generates an inactive shell—likely composed of lithium oxide—at the interface. This interpretation is corroborated by the already discussed cryo‐TEM analysis (Figure S11). Upon Li stripping, this shell remains at the interface. However, its poor adhesion to the CC can lead to interfacial detachment. Given its rigid nature, application of stacking pressure is necessary to preserve contact. This explains why stacking pressure is important for the anode interface.

Simulations were performed to investigate the mechano‐electrochemical process of Li deposition at the interface. The displacement and the corresponding stresses were computed for the linear elastic problem. Stress is a second‐order tensor σ, that can be split into hydrostatic and deviatoric parts. The hydrostatic stress can be computed as σ_0_ = 1/3trace(σ) and is interpreted as the driving quantity for diffusion. We introduce σ_zz_ for the zz‐component of the stress tensor (see methods section for details). Figure 5 presents the simulation results for different assumptions of plastic history (from 0% to 20%, see Methods section for details). Let us first introduce the curves with 0% plastic strain. Figure 5a, f shows a schematic of the Ni CC|Li|LPSC SSE interface with Li deposition (at 2 and 10 MPa, respectively). Figure 5b,g illustrate σ_0_ distribution of the stack with 2 and 10 MPa pressures, respectively, for the assumption of 0% plastic strain. Figure 5d,i represents the corresponding σ_0_ along the top face of the SSE in the cases of 2 and 10 MPa stacking pressures, respectively, as indicated by the green line in Figure 5b,g. An inhomogeneous stress state is observed under a stacking pressure of 2 MPa (Figure 5b,d), with the mean value approximately ‐1.2. Specifically, a compressive stress of around 6 MPa is retrieved to the top face of the SSE in the hill area of Li, whereas a tensile stress of approximately 1.8 MPa is found around the periphery of the Li hill, with stress decreasing outward from the hill center. This indicates that diffusion will be unevenly distributed. Therefore, a bump rises due to the deformation of CC by the Li hill, as shown in Figure 4b,f. Such a Li deposition behavior is harmful to the electrochemical performance. A very high current density will be locally concentrated on the tip of the Li‐hill, leading to Li dendrite formation. In contrast, for 10 MPa, a homogeneous stress state over a large part of the Li island could be found (Figure 5g,i), with a sharp stress difference at the boundary area. The larger mean value of around ‐4.8 MPa (with a largest magnitude stress of ∼21 MPa at the narrow boundary region) will act as a driving force facilitating diffusion over a large part of the Li layer. This Li layer was found to have a slope corresponding to the periphery area of the Li layer (Figure S17).

**FIGURE 5:**
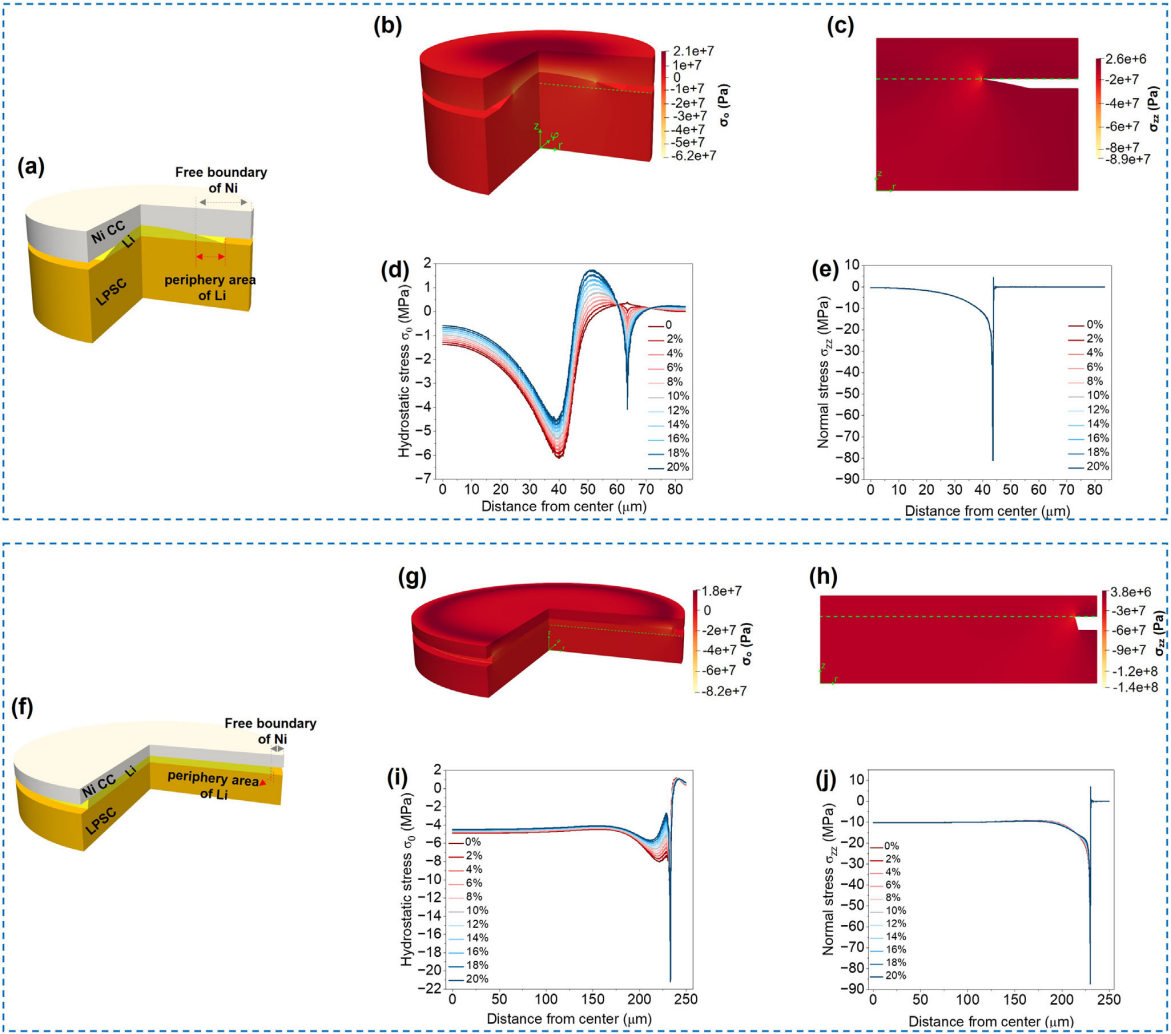
Schematic diagram showing the Ni CC/Li/SSE interface under (a) 2 MPa and (f) 10 MPa stacking pressure. (b) and (g) show hydrostatic stress (σ_0_) distribution for Li islands and vertical pressure on the top boundary for 2 and 10 MPa, respectively. The radius of Li islands under 2 and 10 MPa is adopted to be ∼60 and ∼230 µm, respectively. (c) and (h) present the stress component σ_zz_ distribution for the cases at 2 and 10 MPa. (d) and (i) show the σ_0_ distribution for the 2 MPa case and 10 MPa case, where Li island precipitates along the top face of the SSE, considering the influence of plastic history (from 0% to 20%) in the elastic simulations. (e) σ_zz_ distribution for the 2 MPa case and (j) for the 10 MPa case, showing Li islands on the bottom face of the Ni CC, with the consideration of the influence of plastic history (from 0% to 20%) in the elastic simulations.

Figure 5e,j reports the zz‐components (σ_zz_) along the bottom face of the Ni CC in the cases of 2 and 10 MPa stacking pressures, respectively (along the green line in Figure 5c,h). For 2 MPa case, around the center area, the stress component σ_zz_ is close to zero because the strong buckling of the Ni CC produces a tensile stress that works against the applied pressure. Towards the edge of the Li hill, a rise of σ_zz_ is observed that exceeds the applied pressure by a factor of ≈ 38 before dropping down to zero at the free boundary of the Ni CC. Thus, critical points at which failure of the Ni CC might occur can be identified at the rotation axis as well as the corners of the Li hill. If the Ni CC is not strong enough to withstand σ_zz_, the CC will break, and the Li hill will penetrate the CC layer, similar to the cases reported by Motoyama et al. [40]. Figure 5h shows the σ_zz_ distribution in a cross‐section for 10 MPa. The corresponding plot along the bottom face of the Ni CC is given in Figure 5j. Here, the stress is mostly equal to the applied pressure of 10 MPa before magnifying at the corner with a factor of only ≈14, much smaller than that at 2 MPa.

Besides studying purely elastic scenarios, we introduced an eigenstrain in the Li island region to model plastic history in the simulated snapshots. As we do not know the true plastic history of the Li island, different assumptions of the eigenstrain are presented in Figure 5d,i,e,j. For both the 2 and 10 MPa cases, we do not observe large deviations in σ_zz_, as the distribution is dominated by equilibrium with the applied stress, rather than local plastic strains. In contrast, for the hydrostatic stress σ_0_, different magnitudes of plastic strain produce deviations in the distribution, such as smaller absolute compressive stress minima, larger tensile stress maxima, and more prominent boundary peaks. However, the qualitative course is not significantly influenced. Thus, the predicted diffusion can be considered independent of plastic deformation.

Regions with high local stress are potential sites for the initiation of mechanical damage, which may degrade the mechanical properties of the material. This mechanism may contribute to the limited reversibility of repeated Li deposition in AFSSBs. The stress state below a small island under 2 MPa, shows perturbations induced by Li deposition that extend across the entire island (Figure 5d). For comparison, the stress field below a large island under 10 MPa remains largely homogeneous, with only localized perturbations near its periphery (Figure 5i). This more homogeneous stress state is consistent with the experimentally observed improvement in the uniformity of Li deposition at 10 MPa.

## 3 Conclusion

In conclusion, the critical role of stacking pressure in AFSSBs has been comprehensively investigated by combining electrochemical measurements in a 3‐e cell set‐up, post‐mortem morphological analyses by SEM, FIB and Cryo‐TEM, and mechanical simulations. The 3‐e cell set‐up allowed to separately investigating the influence of stacking pressure on anode and cathode interfaces. The results show that stacking pressure could effectively improve the performance at both anode and cathode interfaces. However, it definitely has a much larger impact on the anode interface, possibly due to the creep/plastic deformation behavior of Li metal. Morphological studies by SEM/TEM also support the creep behavior of Li metal anode under different stacking pressures, while mechanical simulation results suggest that larger deposited Li islands allow for more homogeneous diffusion. This work unveils the critical role of stacking pressure for AFSSBs and provides a deeper understanding of the interface properties in AFSSBs.

## 4 Methods

### 4.1 Preparation of LiNi_0.8_Mn_0.1_Co_0.1_O_2_ Composite Cathode

LiNbO_3_ (1wt.%) coated LiNi_0.8_Mn_0.1_Co_0.1_O_2_ (NMC811@LNO) was used as cathode active material (CAM) and purchased from MSE Supplies. Li_6_P_5_SCl (LPSC) solid electrolyte was purchased from NEI Corporation. NMC811 composite cathode is prepared by dry mixing 70 wt.% NMC811@LNO powders with 27.5 wt.% LPSC powders, 1 wt.% super C65 nano carbon black (TIMCAL) and 1.5 wt.% carbon fibers (VGCF; Sigma–Aldrich, 98% carbon‐base).

### 4.2 Assembling of Anode‐Free Solid‐State Batteries

2‐electrode type anode‐free solid‐state cells were assembled by firstly pressing 170 mg LPSC powders in a 12 mm diameter size CompreCell 12 from rhd Instruments under 80 MPa for 1 min using a CompreDrive (rhd Instruments). Then, two stainless steel pillars from both sides were taken out. A 12 mm diameter, 15 µm thick Ni foil was placed on one face of the LPSC SSE pellet. On the other face of the LPSC SSE pellet were spread with 11 mg of the NMC811 composite cathode powders. After that, the two stainless steel pillars were put back in CompreCell 12, followed by the application of 370 MPa pressure for 1 min to densify the solid‐state batteries. After densification, the pressure was reduced to 0, 2, 5, 10, or 20 MPa for electrochemical testing.

For assembling the 3‐e anode‐free solid‐state cell, a RE with Li coated Ni wire was used. The Li coated Ni wire was prepared by dipping a 100 µm‐sized Ni wire into molten Li (at 280°C) for 5 s. Anode‐free solid‐state cells with REs were built using CompreCell 12‐DP. The cell setup could be found at rhd‐instruments website [42]. Firstly, the Li coated Ni wire was located at the middle of the cell and penetrated through the cylindrical PEEK sleeve. Some parts of Ni wire were exposed outside of the cylinder, for contacting with the cable. One stainless steel rod was first placed on one side of the cylinder, with around 1 mm below the wire. Then, 300 mg of LPSC powders were inserted into the cylinder with another stainless steel rod applied into the cell. A 80 MPa pressure was then applied to densify the LPSC powders. After that, two stainless steel rods from both sides were taken out. A 12 mm diameter, 15 µm thick Ni foil was placed on one face of the LPSC SSE pellet. On the other face of the LPSC SSE pellets, 11 mg of NMC811 composite cathode was placed. Then, the two stainless steel rods were put back into the CompreCell 12 [43], followed by 370 MPa pressure for 1 min to densify the cell stack. After densification, the pressure was reduced to 2, or 10 MPa for electrochemical testing.

### 4.3 Electrochemical Testing

Anode‐free solid‐state cells were tested by ModuLab XM ECS (Solartron Analytical / Ametek). 2‐e and 3‐e anode‐free cells were all cycled at 0.1 C (1 C = 180 mAh g^−1^), with charging/discharging cut‐off voltages of 2.5 – 4.25 V, at room temperature. Electrochemical impedance spectroscopy (EIS) was performed at frequencies of 1 MHz to 0.1 Hz for 2‐e cells, and 300 kHz to 1 Hz for 3‐electrode cells.

### 4.4 Materials Characterization

Scanning electron microscope (SEM) imaging coupled with FIB and Energy‐Dispersive X‐ray Spectroscopy (EDX) was performed using a ZEISS Crossbeam XB340. For observing the surface and cross‐sectional morphologies of Li plating and stripping at the Ni current collector and LPSC SSE interface, the cell pellets were taken out from the CompreCell at the desired state of charge/discharge. To avoid the deformation of the Li during the pellet removal process, the cells were moved out by applying force from the cathode side. For surface morphology study by SEM, Ni current collectors were peeled off from the SSE. For a cross‐sectional morphology study, a sharp blade was used to cut the cell from the Ni foil surface to expose the cross‐sectional view. Cryo‐condition transmission electron microscope (Talos F200i) with scanning transmission electron microscopy (STEM) was performed as well. For preparing a cryo‐condition TEM sample, anode‐free solid‐state cells were first assembled. A 3 mm diameter Ni grid was placed between the Ni foil and the LPSC SSE. After charging for 30 mAh g^−1^ of capacity at 10 MPa, the cell was disassembled, and the Ni grid was taken out for Cryo condition TEM study. All the processes were performed under an inert environment.

### 4.5 Mechanical Simulations

Two scenarios were simulated with mechanical models: 2 MPa stacking pressure with a small Li island and 10 MPa stacking pressure with a large Li island. For the simulation, the battery is considered as a solid with three domains of different materials according to Figure 5a,g. The system is assumed axially symmetrical, for which a cylindrical coordinate system (r,φ,z) was used. The material properties are assumed isotropic and linear elastic in each domain, with the respective parameters given in Table S1. As the applied stresses are above the yield stress of Lithium (0.57–1.26 MPa) [44], we model an additional eigenstrain in the island domain that represents plastic history. With that, the underlying material law becomes:

$$\sigma =C\;\left(\varepsilon -\varepsilon^{p}\right)$$

where σ is the second‐order Cauchy stress tensor, C the fourth‐order stiffness tensor, and ε the second‐order infinitesimal strain tensor. The eigenstrain tensor ε^p^ represents plastic history and is therefore constructed trace‐free:

$$\varepsilon^{p}=\left(\begin{array}{ccc} 0.5f & 0 & 0\\ 0 & 0.5f & 0\\ 0 & 0 & -f \end{array}\right)$$

where we introduce f as a fixed absolute value of plastic strain. The elastic problem is formulated with the following boundary conditions:

- Zero vertical displacement of the bottom boundary;
- zero radial displacement of the axis at r = 0;
- vertical pressure on the top boundary of p = 2 MPa for the small Li island and p = 10 MPa for the large Li island.

A finite element model is built with the finite element library of the FEniCS Project [45, 47, 48]. The mesh was generated with the mesh generator GMSH [49]. We compute the displacement and the corresponding stresses for the elastic problem. For instance, component σ_zz_, is the stress acting in the z‐direction. We are furthermore interested in the hydrostatic stress σ_0_, which is linked to purely volumetric deformations, i.e. the widening of the crystal lattice. It can be computed from the stress tensor by:

$$\sigma_{0}=\frac{1}{3}\;trace\;\left(\sigma \right)=\frac{1}{3}\;\left(\sigma_{rr}+\sigma_{\varphi \varphi }+\sigma_{zz}\right)$$

Diffusion is attracted into areas with tensile hydrostatic stress due to a local volume extension of the lattice. Consequently, areas with large compressive hydrostatic stress act as the sources for the diffusion. Therefore, we can interpret σ_0_ to assess the diffusion behavior of Li in the cell.

However, the present model does not explicitly resolve the diffusion process as this would exceed the scope of the simulations. The approach allows only for an approximation of the precipitation, under several limitations. First, the coupling between stress and diffusion is not captured, as no governing transport equation (e.g., stress‐driven diffusion) is solved. Second, the temporal evolution of the system is not resolved: the parameter f does not correspond to a physical time scale, but merely parameterizes different assumed states of plastic deformation. Consequently, the model provides a tool to simulate snapshots rather than a true kinetic description of the process. Despite these limitations, the approach enables efficient exploration of the mechanical response of the system under varying assumed precipitation states and loading conditions, without incurring the significant computational cost associated with fully coupled diffusion‐mechanics simulations.

## Author Contributions

**Jianneng Liang**: conceptualization, investigation, writing – original draft, writing – review and editing, validation, methodology, data curation. **Shuya Gong**: methodology, investigation, data curation. **Mervyn Rohit Soans**: methodology, investigation, writing – review and editing. **Alberto Varzi**: conceptualization, supervision, funding acquisition, resources, writing – review and editing. **Stefano Passerini**: writing – review and editing, supervision. **Robert Leiter**: methodology, data curation, investigation, formal analysis, writing – review and editing. **Simon Fleischmann**: methodology, supervision, writing – review and editing, funding acquisition. **Matthias Bohnen**: software, data curation, methodology, formal analysis, writing – review and editing. **Ralf Müller**: methodology, software, formal analysis, writing – review and editing.

## Funding

This work was supported by Federal Ministry for Economic Affairs and Energy (BMWE) in the frame of the REFA (03EI6055B) and ELIC (03ETE034C) projects.

## Conflicts of Interest

The authors declare no conflicts of interest.

## Supporting information




**Supporting File 1**: advs76888‐sup‐0001‐SuppMat.docx.

**Supporting File 2**: advs76888‐sup‐0002‐DataFile.zip.

## Data Availability

The data that support the findings of this study are available from the corresponding author upon reasonable request.
