# Supplementary material for: Stacking Pressure‐Driven Interfacial Dynamics in Anode‐Free Solid‐State Lithium Batteries
Source: Adv Sci (Weinh). 2026 Aug 5;13(59):e76888. doi: 10.1002/advs.76888 (PMC13440368; doi:10.1002/advs.76888)
Supplement: Supplementary file 1 [file ADVS-13-e76888-s002.docx]

Supporting Information

Stacking Pressure–Driven Interfacial Dynamics in Anode-Free Solid-State Lithium Batteries

Jianneng Liang*, Matthias Bohnen, Ralf Müller, Robert Leiter, Simon Fleischmann, Shuya Gong, Mervyn Rohit Soans, Stefano Passerini, Alberto Varzi*

Dr. J. Liang

School of Resources, Environment and Materials, Guangxi University, 530000 Nanning, China

E-mail: [jliang95@gxu.edu.cn](mailto:jliang95@gxu.edu.cn)

Dr. J. Liang, Dr. R. Leiter, Dr. S. Fleischmann, S. Gong, Dr. M. Rohit Soans, Dr, A. Varzi

Helmholtz Institute Ulm (HIU) Electrochemical Energy Storage, Helmholtzstrasse 11, 89081 Ulm, Germany

E-mail: [alberto.varzi@kit.edu](mailto:alberto.varzi@kit.edu)

Dr. J. Liang, Dr. R. Leiter, Dr. S. Fleischmann, S. Gong, Dr. M. Rohit Soans, Dr, A. Varzi

Karlsruhe Institute of Technology (KIT), P.O. Box 3640, 76021 Karlsruhe, Germany

M. Bohnen, Prof. Dr.-Ing. R. Müller

Institut for Mechanics, Department of Civil and Environmental Engineering, TU Darmstadt

Prof. S. Passerini

International Electrochemical Energy Storage Research Institute (IEES)

School of Energy and Mechanical Engineering Nanjing Normal University Nanjing 210023, China

**Figure S1** Nyquist plots of AFSSBs under different stack pressures.

**Figure S2** First-cycle cell voltage versus capacity profiles of AFSSBs under different stack pressures. For the 0 MPa AFSSB, only the charge profile is presented as the discharge step could not be performed due to cell failure.

**Figure S3** Cell voltage vs. capacity profile of AFSSB at 20 MPa stacking pressure.

**Figure S4** cell configuration in three-electrode mode to enable the measurement of cathode, anode and full cell potentials, and impedances.

**Figure S5** Equivalent circuits for fitting the Nyquist plots of anode and cathode. R_SSE_ represents the electrolyte resistance. R*_SEI_* (R_CEI_) and CPE*_SEI_* (CPE*_CEI_*) represent the resistance and capacitance associated with the solid electrolyte interphase (SEI) (or cathodic electrolyte interphase (CEI)). R*_ct_* and CPE*_ct_* represent the resistance and capacitance associated with the charge transfer.

**Figure S6** Cycling performance of AFSSBs under different stacking pressure.

**Figure S7** Evolution of anode potential vs. Li/Li^+^ during a full rest, charge, rest, and discharge cycle at 2 MPa stack pressure. The insert is the anode potential between 71-75 h.

**Figure S8** Evolution of anode voltage vs. Li/Li^+^ during a full rest, charge, rest, discharge cycle at 10 MPa stack pressure. The insert is the anode potential between 207-210 h.

**Figure S9** 2 MPa full cell EIS Nyquist plot of Ni/SSE/NMC811 AFSSB at the end of charge (a) and at the end of discharge (b) for the first 10 cycles.

**Figure S10** 10 MPa full cell EIS Nyquist plot of Ni/SSE/NMC811 AFSSB at end of charge (a) and at the end of discharge (b) for the first 10 cycles.

**Figure S11** Cryo-TEM images of Ni CC and SSE interface. (a) Bright-field-TEM image and (b) dark-field-TEM image of the Li island growing from a SSE particle surface. (c) Electron diffraction of an area from (b). The diffraction patten indicating the formation of Li_2_O nano particles. (c) EELS spectra from the marked spot shows clear Li K edge with onset at 55 eV (d), but no clear S L2/3 edge at 162 eV (e).

**Figure S12** (a) A rod-structured Li metal grown at the interface between Ni grid and SSE particle. (b) The corresponding HADDF STEM image at the selected area in (a). (c) Li EELS mapping of corresponding area. (d) Image of an area containing LPSC SSE and Li rod and (e) corresponding EDX mapping. The area corresponding to Li contains O but no S.

**Figure S13** (a-e) surface SEM images of Ni CC after charging at AFSSBs under 0, 2, 5, 10, and 20 MPa. (f-j) Under each SEM image, a schematic illustration shows the Li deposition status.

**Figure S14** Li deposit on Ni CC surface tested at 10 MPa after (a) 4 h, (b) 5 h and (c) 10 h deposition a current density of 0.12 mA cm^-2^.

**Figure S15** Ni/Li/SSE interface study at the end of charging at 10 MPa. (a) cross-sectional SEM images of Ni/Li interface after FIB cut. (b) schematical diagram showing the interface structure and Li deposition behavior. Due to the particle structure of SSE powders, Li deposition between Ni/SSE interface will result in some SSE powders buried inside Li island. (c) a selected SEM area for EXD mapping. (d)~(h) corresponding EXD mapping result of (d) summary, (e) Ni, (f) O, (g) S, and (h) P. EDX could not identify Li. However, due to the high reactivity of Li with Oxygen, we usually used O element distribution to represent the Li distribution.

**Figure S16** Ni/Li/SSE interface study at the end of discharging state at 10 MPa. (a) cross-sectional SEM images of Ni/Li/SSE interface after FIB cut. (b) a selected SEM area for EXD mapping. (c)~(f) corresponding EXD mapping result of (c) summary, (d) O, (e) S, (f) P. EDX could not identify Li. However, due to the high reactivity of Li with Oxygen, we usually used O element distribution to represent the Li distribution.

**Figure S17** Cross-section image of CC/Li/SSE interface (tested at 10 MPa stack pressure) at the end of the charge. Charging for 7.7 h, at a current density of 0.12 mA cm^-2^.

**Table S1** Properties of the computation domain

| **Materials** | **Li** | **Ni** | **Li_6_PS_5_Cl** |
| --- | --- | --- | --- |
| Young’s Modulus (GPa) | 7.82 | 190 | 21 |
| Poisson’s Ratio | 0.381 | 0.305 | 0.28 |
